# Mitochondrial Genomes of Korean Native Black Goats Reveal Shared Phylogeographic Patterns and Demographic History

**DOI:** 10.3390/ani14202949

**Published:** 2024-10-13

**Authors:** Gaeun Kim, Eundo Lee, Kwanwoo Kim, Dongkyo Kim, Seungchang Kim, Daehyeok Jin, Huimang Song, Seongsil Mun, Hankyeol Jeong, Jaemin Kim, Bonghwan Choi

**Affiliations:** 1 Animal Genetic Resources Research Center, National Institute of Animal Science, Rural Development Administration, 224, Hamyang 50000, Republic of Korea; gaeun0210@korea.kr (G.K.);; 2 Division of Animal Bioscience & Integrated Biotechnology, Gyeongsang National University, Jinju 52828, Republic of Korea; 3 Division of Applied Life Science (BK21), Gyeongsang National University, Jinju 52828, Republic of Korea; 4 Institute of Agriculture and Life Sciences, Gyeongsang National University, Jinju 52828, Republic of Korea

**Keywords:** Korean native black goats, mitochondrial DNA, phylogenetic analysis, origin, genome

## Abstract

**Simple Summary:**

This study explores the phylogenetic relationships among Korean native black goats, whose genetic resources are being preserved by the National Institute of Animal Science through the collection of purebred specimens. We analyzed the complete mitochondrial genomes of 282 native goats and 82 reference populations. The native population exhibits a haplotype diversity of 0.659 and includes 39 distinct haplotypes. Neighbor-joining tree and median-joining network analyses established that these goats form a unique clade (A’) within Haplogroup A, suggesting a shared ancestry with diverse goat populations from around the world.

**Abstract:**

This study explores the phylogeny of Korean native black goats through analysis of their complete mitochondrial DNA. The National Institute of Animal Science has gathered genetic material on purebred goats from isolated regions such as Tongyeong, Dangjin, and Jangsu, and is actively breeding them on a national level. These populations, however, are small and exhibit high inbreeding rates, highlighting the urgent need to preserve genetic diversity. The haplotype diversity within this native group is 0.659, with 39 haplotypes identified. By contrast, including international breeds in the analysis increases the overall haplotype diversity to 0.925 with 203 haplotypes identified, highlighting the limited genetic diversity among native black goats. For phylogenetic assessment, a neighbor-joining tree and median-joining network were constructed using identified haplogroups (A, B, C, D, G, and F) from prior studies. The results pinpoint the native black goats as closely related to, but distinct from, Haplogroup A with a bootstrap value of 98, establishing them as a separate clade (A’). This supports the notion of a shared ancestry with various global populations. This research provides essential data on the origins and evolutionary history of Korean native black goats, supporting conservation and breeding efforts aimed at enhancing genetic diversity.

## 1. Introduction

Goats are among the earliest domesticated animals, with their wild ancestor being Capra aegagrus, known as the bezoar [1,2,3]. Debate surrounds the origins of the domesticated goat (*Capra hircus*), but it is generally believed that they first appeared in the Fertile Crescent. Archaeological findings suggest that goat domestication began around 10,000 years ago in the Euphrates River area of southeastern Anatolia [4]. Known as “the poor man’s cow”, domesticated goats provide both meat and milk and are adaptable to diverse environments including tropical regions, deserts, temperate zones, and cold mountainous areas, which makes them one of the most widespread livestock species globally [5].

The native black goat, a highly adaptable breed, demonstrates good productivity in Korea, where more than 70% of the landscape is mountainous. This breed, distinguished by its black or dark brown color, horns, and beards without fleshy growths, is the sole black-colored goat breed on the Korean Peninsula [6,7]. At 12 months of age, these goats typically weigh between 21–25 kg. Currently, ~450,000 native black goats are raised in Korea. The National Institute of Animal Science has been collecting purebred populations since 1994 in Bunam-myeon, Jangsu-gun, Jeollabuk-do, and since 1997 on islands like Yokjido in Tongyeong, Gyeongsangnam-do, and Anmyeon-do in Taean-gun, Chungcheongnam-do [8]. The Department of Animal Science, at Gyeongsang National University, have also gathered purebred populations, which are maintained through institutional and university exchanges.

Currently, the National Institute of Animal Science is raising about 140 purebred native black goats, making efforts to preserve and expand the population by line. Each line has distinct characteristics: the Jangsu line has shorter hair and is similar to the Tongyeong line but has a higher mature body weight. The Dangjin line has longer hair compared to other lines and particularly shows a dark brown color, especially in male goats, with faster growth. The Tongyeong line has short hair, and both males and females exhibit a lower growth rate and mature body weight. Due to the small numbers in each line, a temporary goat breeding program developed by the government is being tested to reduce inbreeding coefficients. It is expected that farmers will be able to utilize this program in the future when the goat industry becomes more active, and a traceability system is implemented. Furthermore, protocols for artificial insemination and estrus synchronization have been developed to manage the reproduction of seasonally breeding goats [9], and ongoing research is being conducted to increase fertility rates to expand the population.

The origin of this goat is traced back to the early Han Dynasty (A.D. 25–219), as described in Liu Xi’s book “Shiming”, which references “Hanyang (韓羊), Hanto (韓兔), and Hangyeo (韓鷄)”. This suggests the presence of a distinct type of sheep during the latter part of the Three Hans period (B.C. 300–A.D. 300). ‘Han’ in this context likely refers to Mahan, which includes the regions of present-day Chungcheong and Jeolla in South Korea, suggesting an introduction from the north. However, there are no records of sheep in the northern part of the Korean Peninsula, and they are not mentioned in the official titles during the Buyeo period (B.C. 400–A.D. 494), implying they were not a significant part of the livestock. Archaeological findings at the first-century Gimhae shell mound site include deer, cattle, and horse remains but no sheep or goat bones, further suggesting that these animals were not present in the region at that time. These findings suggest that goats were likely introduced to Korea through the western coastal regions of the southern peninsula from China [8].

To accurately understand the lineage and origin of native black goats, mitochondrial genome analysis is necessary. Mitochondrial genomes evolve faster than nuclear DNA, which makes them valuable for detailed evolutionary studies [10]. Previous phylogenetic studies that have used mitochondrial DNA have been limited to specific geographical regions, and given that each gene region within the mitochondrial genome has the potential to evolves at a different rate, a comprehensive analysis using the entire mitochondrial genome sequence is crucial [11]. In this study, we aimed to investigate the genetic origins and phylogeny of Korean native black goats by analyzing the complete mitochondrial sequence. This approach allows for a more comprehensive understanding of the genetic diversity and evolutionary history of these goats, which have been relatively understudied compared to global breeds. By focusing on the full mitochondrial genome, we sought to uncover more precise phylogenetic signals and determine the position of the Korean native black goats within the broader haplogroup classifications, contributing to the conservation of their unique genetic heritage.

## 2. Materials and Methods

### 2.1. Sample Collection and DNA Extraction

We collected 364 samples from 10 different breeds, including Nubian, Alpine, Saanen, Toggenburg, Boer, various populations of the Korean native black goat (KD; Dangjin, KT; Tong-Yeong, KJ; Jangsu, KG; Gyeong-Sang Uni., KC; Crossbred), and reference populations (PopSet, GenBank 906344678, *n* = 82). The Korean native black goats from Dang-jin, Tong-Yeong, and Jang-su are purebred populations sourced from isolated regions and preserved by the institution, while the Gyeong-Sang Uni. population was collected by Gyeongsang National University. The samples (*n* = 282), primarily blood, were taken from both farms and individuals reared within the institution. DNA extraction was performed using the Promega kit (Promega Corporation, Madison, WI, USA). Genomic DNA was extracted from whole blood using either 300 µL or 3 mL sample volumes. Blood samples were mixed with Cell Lysis Solution to lyse red blood cells, followed by centrifugation to pellet white blood cells. After removing the supernatant, the white blood cell pellet was resuspended and lysed with Nuclei Lysis Solution. Protein was precipitated using Protein Precipitation Solution and removed via centrifugation. The supernatant containing DNA was mixed with isopropanol to precipitate the DNA, which was then washed with 70% ethanol. The DNA pellet was air-dried and rehydrated with DNA Rehydration Solution before storage at 2–8 °C. DNA concentration was determined using a NanoDrop device (Thermo Fisher Scientific, Waltham, MA, USA) [12], and a working solution was prepared at a concentration of 10 ng/µL for experimental use.

### 2.2. DNA Amplification and Sequencing

The complete mitochondrial DNA sequence for *Capra hircus* was targeted with a sequence set (PopSet, GenBank 906344678, *n* = 82) sourced from GenBank via NCBI(National Center for Biotechnology Information, Bethesda, MD, USA). Primer sequences were designed using the NCBI Primer Blast program, resulting in 16 pairs of primers for amplification of circular mitochondrial DNA (Table 1), ensuring overlapping ends of amplified fragments. The PCR reaction mixture included 2 µL 10× buffer, 1.6 µL 10 mM dNTP mix, 0.4 µL forward primer (10 pmol/µL), 0.4 µL reverse primer (10 pmol/µL), 0.2 µL Taq polymerase (2.5 U/µL), 13.4 µL distilled water (DW), and 2 µL DNA (10 ng/µL), creating a final volume of 20 µL for template DNA amplification. PCR conditions were set at 95 °C for 15 min, followed by 35 cycles at 94 °C for 40 s, 60 °C for 40 s, 72 °C for 40 s, and a final extension at 72 °C for 10 min. PCR products underwent purification using MultiScreen HTS Filter Plates(Merck Millipore, Burlington, MA, USA) [13] and 96-well plates. For sequencing, 3 µL purified DNA was used for cycle sequencing with both forward and reverse primers. A second purification was performed prior to sequencing on the ABI 3500 Genetic Analyzer(Thermo Fisher Scientific, Waltham, MA, USA) [14], following the BigDye protocol.

### 2.3. Data Analysis

The sequences were manually edited and curated using BioEdit 7.2 software [15]. Alignment was performed with the Clustal W algorithm in MEGA v.11 [16]. Phylogenetic trees were constructed using the neighbor-joining (NJ) method [17], and tree reliability was assessed via bootstrap analysis with 1000 replicates, setting a bootstrap threshold of 60%. Haplotype diversity (HD), nucleotide diversity (π), and average pairwise differences (k) were calculated using DnaSP version 5.10.1 [18]. Tests for neutrality, including Tajima’s D and Fu’s tests, were conducted to evaluate the neutrality of the sequences. Relationships between populations were analyzed through a median-joining (MJ) network using PopArt software 1.7 [19]. Python 3.12.4 was used to handle missing data in the dataset [20], and R version 4.4.1 was used to generate stability plots and mixture bar plots to assess the stability of the K value and population structure [21]. Mismatch distribution analysis was also conducted using Python. For a comprehensive comparison, plots combining data from native and foreign breeds with reference sequences were created. A Bayesian skyline plot (BSP) was generated using BEAUti and BEAST version 2.7.7 [22]. The general time reversible (GTR) site model was selected in BEAUti, using a Strict Clock model. A generation interval of 1.5 years and a mean clock rate of 3.95 × 10^−8^ substitutions per nucleotide per year (one mutation every 1522 years) were applied, based on previous studies [23]. The chain was run for 10,000,000 iterations, with logs saved every 3000 iterations, and the results were visualized in Tracer v1.7.2 [24].

## 3. Results

### 3.1. Genetic Diversity and Structure Analysis

#### 3.1.1. Haplotype Diversity and Statistical Analysis

We analyzed 364 complete mitochondrial sequences, including 282 from preserved and collected native black goats and foreign breeds, along with 82 reference sequences. The reference sequences included 81 from a PopSet dataset, previously published in a study on goat haplogroups [23], and one representative mitochondrial DNA sequence of Capra hircus from the NCBI database.

Analyses of haplotype diversity (HD), nucleotide diversity (π), and the mean number of nucleotide differences (k) were conducted for Korean native black goat populations (KD, KJ, KT, KG, KC) and foreign breeds (Nubian, Alpine, Toggenburg, Saanen, Boer, Ref) (Table 2). HD values among the Korean populations ranged from 0.608 to 1.000, suggesting that while the crossbred population (KC)’s HD (0.897 ± 0.056) is high, it is not exceptional. In comparison to previous studies, which reported HD values of over 0.97 in 39 out of 54 countries surveyed [1], all of the foreign breeds we sampled (Nubian, Alpine, Toggenburg, Saanen, Boer) recorded values above 0.97, with the exception of Boer. In contrast, even though KC had the highest HD value among the Korean native black goats, it still fell short of 0.97, indicating a relatively lower genetic diversity. However, KC’s π (0.02061) stands out, indicating genetic mixing. Despite the relatively high π, the population displayed a low k, reflecting small population size and possible isolated breeding, which may lead to reduced overall genetic diversity.

By comparison, the Alpine (1.000 ± 0.096) and Nubian (1.000 ± 0.500) breeds showed the highest HD, with the Alpine breed also exhibiting a notably high π (0.00323). The Korean native populations (*n* = 161) exhibited 68 variable sites, whereas the Alpine breed (*n* = 6) had 103, highlighting the genetic homogeneity within the Korean native black goat populations.

In terms of the number of variable sites across different breeds, the native goats (*n* = 161) had only 68 sites compared to the 103 sites in the Alpine breed (*n* = 6), which had the highest genetic diversity. This contrast highlights the significant genetic homogeneity within the Korean native black goat populations compared to their number of individuals.

Tajima’s D, a measure used to assess the neutral evolution of genes, yielded a value of −1.81616 for the native population. Typically, a negative value suggests positive selection, population expansion, or genetic hitchhiking. Given that the *p*-value was <0.05, this Tajima’s D value was deemed statistically significant [25].

Fu’s Fs statistic was −11.253, suggesting population expansion or genetic hitchhiking. This markedly negative value, along with the Tajima’s D value of −1.81616, indicates deviations from neutral evolution. Together, these results strongly support the hypothesis that the Korean native black goat populations have undergone recent population expansion or have been subject to positive selection. These findings provide important insights into the evolutionary processes shaping the genetic structure of the population [25].

#### 3.1.2. Study of Population Structure Analysis

**Figure 1:**
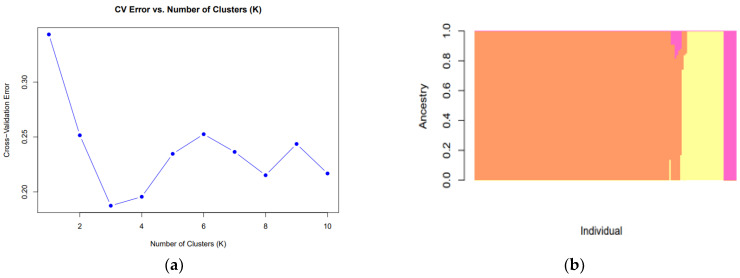
Cross-validation graph of K values (**a**) and Population structure analysis (**b**) using whole mitochondrial sequences of Korean native black goats (*n* = 144).

The cross-validation graph for varying numbers of clusters (K) showed a sharp decrease in error transitioning from K = 1 to K = 3, followed by a minor increase at K = 4(Figure 1a). The optimal number of clusters was identified as K = 3, showing the lowest error value. This explains the use of three putative ancestors in the population analysis and their application in the admixture analysis. The analysis included 144 native black goats (KD, KT, KJ, KG) and indicated that K = 3 provided the most stable solution. This may be due to the small sample size of the KG group (*n* = 2) or its genetic similarity to other populations, which likely contributed to the outcome.

We excluded crossbred animals (KC) from the admixture analysis to focus on the genetic structure and ancestry of the purebred native black goat populations. The population structure analysis revealed distinct and homogeneous ancestral patterns(Figure 1b). Certain individuals showed a clear dominance of a single cluster, while others exhibited mixed ancestry. These findings suggest that classifying the population structure at K = 3 adequately captures the genetic structure of the breed, indicating the presence of three distinct lineages within this breed.

#### 3.1.3. Mismatch Distribution and BSP

Mismatch distribution analysis and BSP were conducted separately for the native group (b, *n* = 144) and the entire group including these goats (a, *n* = 364) to obtain objective insights by contrasting the native population against global haplotypes.

**Figure 2:**
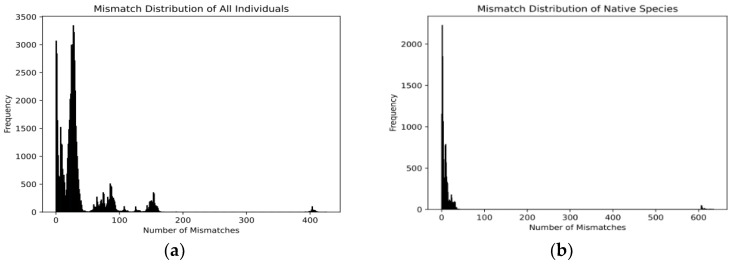

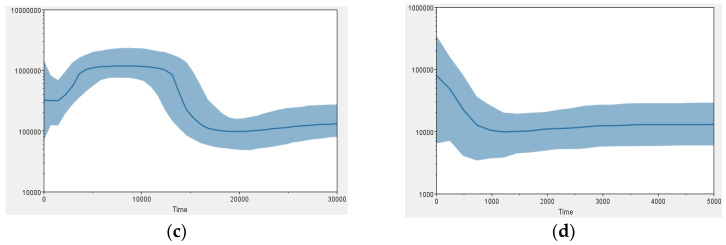
Mismatch distribution ((**a**) all individuals; (**b**) native species) and BSP ((**c**) all individuals; (**d**) native species) of population expansion. In the BSP, the *y*-axis represents effective population size (Ne), and the *x*-axis represents time (years before present). The blue shaded region indicates the 95% highest posterior density (HPD) interval, and the dark line represents the median Ne estimate.

To explore the demographic history, such as population size changes, expansions, and bottlenecks, we performed mismatch distribution analysis, which measures pairwise differences between DNA sequences (Figure 2a,b). For the entire population, the mismatch distribution is broad (Figure 2a), reflecting a diverse gene pool. The main peak of the distribution is in the range of 0–50 mismatch pair range, indicating that most sequence pairs are very similar, with several peaks of varying sizes, suggesting multiple lineages or populations. The extended tail on the right side of the graph indicates significant genetic divergence among some sequence pairs.

By contrast, the mismatch distribution for the native goats is narrow (Figure 2b), pointing to a more homogenous gene pool with smaller genetic differences among individuals. The main peak is tightly concentrated in the 0–20 range, indicating high genetic similarity within this group. There is no extended tail, signaling minimal genetic heterogeneity within this population.

The BSP provides insights into the historical population dynamics of a species (Figure 2c,d). The *y*-axis represents the effective population size, and the *x*-axis represents time. Data from the entire population (Figure 2c, *n* = 364) and the purebred native population (Figure 2d, *n* = 144) were used.

The expansion period began 11,000 years ago, predating the estimated time for global goat domestication. This finding aligns with previous research [23,26]. When constructing the BSP, the mean clock rate (mutation rate) was 3.95 × 10^−8^ [23]. For a more accurate estimation of the timeline (*x*-value), a mean clock rate accounting for different mutation rates across various regions, such as the HVI region and coding region, is necessary. Therefore, the focus should be on the shape of the plot rather than the precise numerical values.

The plot, for the entire group, depicts a period of stabilization following an initial increase in population size. Integrated with the mismatch distribution analysis, the multiple peaks suggest a history of expansion and contraction events. Conversely, the plot for the native goats shows a recent population increase following a mild initial bottleneck, consistent with the rapid population increase indicated by the mismatch distribution analysis.

### 3.2. Phylogenetic and Network Analysis

#### 3.2.1. NJ Tree

**Figure 3:**
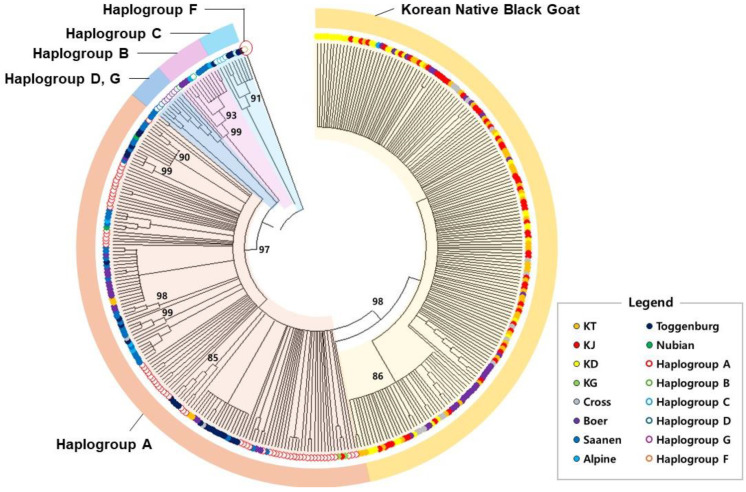
Phylogenetic relationships among 364 individuals. The Korean native black goat is positioned closest to Haplogroup A among the six distinct haplogroups.

NJ trees are widely used in phylogenetic analyses to infer the evolutionary relationships among a series of taxa or sequences. This method uses genetic data to understand the genetic distances between various individuals or populations. To construct the NJ tree, a bootstrap analysis was performed 1000 times on all 364 individuals, with a cut-off value of 60 used to generate the results (Figure 3).

**Figure 4:**
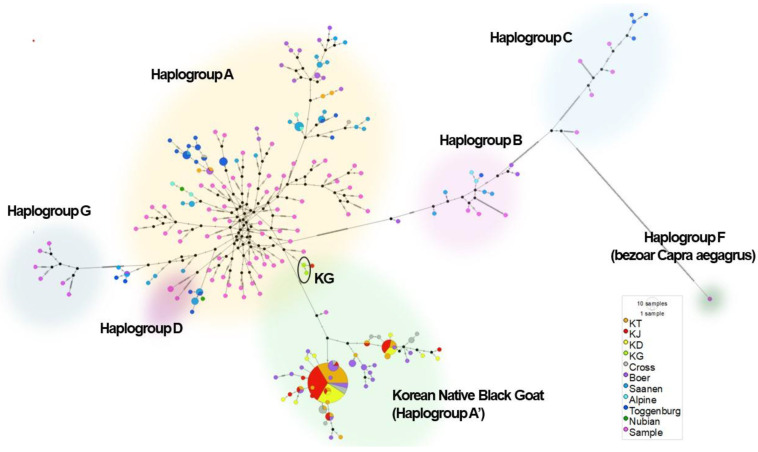
To analyze the maternal phylogenetic structure, an MJ network minimum spanning tree was constructed. In total, 82 reference sequences from NCBI GenBank PopSet were included, representing all Haplogroups (A–G). These reference sequences served as key markers, delineating each haplogroup within the MJ network.

The NJ tree distinctly shows clusters corresponding to Hap A-Hap F. The native goat population is closely associated with Hap A, but forms a separate group from named Hap A with a bootstrap value of 98. However, the native goat population from Gyeongsang National University (KG) belongs to Hap A, not the distinct Hap A’ group (Figure 4).

#### 3.2.2. MJ Network

In the MJ network analysis (*n* = 282, including 82 reference sequences), the goats were categorized into haplogroups A, B, C, D, E, F, and G, with Haplogroup A being predominant globally (Figure 4). Most Korean native black goats (KT, KD, KJ, KC) clustered in Haplogroup A’, while those from Gyeongsang National University grouped with Haplogroup A.

The colored pie charts represent individual samples or populations, each assigned a specific color based on their origin, as shown in the legend. The pie charts for the Korean native black goat (KG), located primarily in Haplogroup A’, feature multiple colors, showing that this group shares genetic similarities with other haplogroups, which highlights its genetic diversity. The size of each pie chart represents the number of samples in each group or the amount of genetic variation within that haplotype. Additionally, the proximity of the Korean native black goat to Haplogroup A suggests a shared ancestry with other global goat populations, while maintaining distinct characteristics within Haplogroup A’.

## 4. Discussion

Phylogenetic analysis can be conducted using either partial or complete mitochondrial sequences. Previously, studies on Korean native black goats have primarily used gene segments including 16s rRNA, cytochrome b, and the D-loop [27]. However, relying on partial mitochondrial sequences may not fully capture the overall genomic trends and could overlook significant evolutionary relationships. Notably, there are distinct differences between paired and unpaired RNA regions: paired regions exhibit low adenine (A) content, high GC content, and slower substitution rates, whereas unpaired regions have higher A content, lower GC content, and faster evolutionary rates [28]. Therefore, we used complete mitochondrial sequences to uncover more precise phylogenetic signals.

A significant finding was determining the specific haplogroup for these goats. Previously, three main haplogroups had been identified; these diverged and expanded ~200,000 years ago [29]. This includes lineages not only from the Fertile Crescent region (Hap A, C) but also from East Asia and South Asia (including Mongolia, Pakistan, India, and Malaysia) as part of Hap B. Subsequent studies led to the reclassification of Hap E under Hap A, the subdivision of Hap B into B1 and B2, and the identification of the distant Hap F group [30,31,32] as well as the Haplogroup G group found near the Fertile Crescent in the Middle East and North Africa [1].

Haplogroup A is predominant worldwide. Our results indicate that Korean native black goats are closest to, but distinct from, Haplogroup A, suggesting they should be considered a separate group (here, A’). These findings highlight the genetic diversity within this population. These goats might have been introduced to Korea either as part of Hap A or already as Hap A’. If they started out as Hap A, they likely experienced a bottleneck and subsequently formed the subgroup. If they arrived as Hap A’, this would imply a distribution of a single haplotype across various regions. However, the proximity of Gyeongsang National University goats (KG) to Hap A suggests that Hap A may have gradually evolved into Hap A’.

A common observation in phylogenetic studies of goats is that, unlike cattle and other livestock, goats exhibit strong gene flow and a weak genetic structure [33]. However, our phylogenetic analysis indicates that all individuals form a branch of Hap A, with no derivatives from Hap B. Historically, Korea had extensive commercial exchanges with neighboring countries, such as China and Japan, and frequent interactions with Southeast Asian countries. In addition, there was a significant trade route, the Silk Road, which stretched from Mongolia through the Middle East and to Europe [34]. Despite these connections, no lineage related to Hap B, which is presumed to have originated close to Hap A, was identified, pointing to the need for further research. Not only the purebred native populations but also the crossbred domestic black goats all belong to Hap A or A’, suggesting that Hap B might have existed in regions with challenging trading conditions or that it has a very brief history. Currently, countries such as China, Pakistan, and India are intensively exploring the origins of their own Hap B lineages [30,31,35]. Future studies should integrate historical trade and commercial dynamics with phylogenetic analyses of Korean native black goats, building on these findings.

## 5. Conclusions

Based on analyses of the entire mitochondrial sequence of Korean native black goats, we find that these goats have lower genetic diversity compared to foreign breeds, a consequence of their small population size and isolated breeding methods. However, crossbred goats exhibit higher genetic diversity. The native goats predominantly belong to the subgroup Haplogroup A’, a subset of Haplogroup A. The presence of Gyeongsang National University black goats, which are closer to Hap A, suggests a gradual transition from Hap A to Hap A’. This may have occurred via population bottlenecks and expansion.

This study also demonstrates the importance of preserving the unique haplotypes of these goats and addressing their low genetic diversity. Furthermore, additional research is needed to understand why these goats today lack Haplogroup B, despite historical trade routes with nearby regions. Collaborative research with countries such as China and Pakistan could provide deeper insights into the migration patterns of goats across Asia.

## 6. Limitations of the Study

One of the primary limitations of this study is the small sample size, particularly in terms of native Korean black goats. While we acknowledge the relatively small number of animals analyzed, significant efforts were made to locate and source purebred native black goats in Korea. The National Institute of Animal Science worked extensively across the country to gather these animals, and the total population size, including the Jangsu, Tongyeong, Dangjin, and Gyeongsang National University lines, is estimated to be approximately 140 individuals. Given the limited availability of these purebred animals, we made every effort to include as many as possible in our study. Additionally, previous studies analyzing purebred goat lineages globally have utilized sample sizes ranging from 10 to 40 individuals, demonstrating that our study is comparable in scale to other genetic research efforts involving indigenous breeds [31,36,37].

Another notable limitation is the potential bias that could arise from the differences in sample size between the groups analyzed, particularly for the Korean G group, which consisted of only two individuals. This imbalance in sample size may affect the generalizability of the results. Moving forward, given that the Korean G group is a small population, future research should aim to increase the sample size by collecting samples across multiple generations to enhance the robustness of the findings.

Finally, in future studies, we aim to include a wider range of Asian breeds, such as those from China and India, to gain a more comprehensive understanding of the origins and migration history of native black goats. Including other Asian breeds will allow for a deeper understanding of the genetic diversity and phylogenetic relationships of native black goats across the region.

## Figures and Tables

**Table 1:** Mitochondrial DNA Primer Sequences for PCR Amplification.

No	Forward Primer	Reverse Primer	Product Length (bp)
1	TAGGTTTGGTCCCAGCCTTC	TGAGTAGCTCGTCTGGTTTCG	1285
2	TGGCGCTATAGAGAAAGTACCG	TCACCCCAACCAAAACTGCT	1062
3	CAGTGAAATTGACCTCCCCGT	GTGCTCGGTTTGTTTCTGCT	1227
4	GCCTTACGAGCAGTAGCACAA	TAGTCCTCCTCAGCCTCCAATTAT	1282
5	AGCTCCATTCCACTTCTGAGTC	TTCGGCGCGAATTAGTAAGC	1219
6	AACCAACCACAAAGACATCGG	TGTTGTGGGAAGAAGGTCATGT	1260
7	TCAATAGGAGCTGTGTTCGCT	GGCCGTAGAATAGACCTGGAC	1127
8	AGACGCAATTCCAGGTCGTTTA	GTAGGAGAGCGGATAGTGCC	1157
9	AGCCAACATCACAGCAGGAC	TAGTTGGGGGAGTCAAAATGCG	1132
10	GCTGCCTGATACTGACACTTTG	GCTCTGTTTGGTTTCCTCATCG	1272
11	ACATTCACCGCTACAGAACTAATTT	AGGGTTAGGGTGGTTAGTGC	1297
12	AACGGGGTAAACATACCCATCA	TGGAGTAATGCTGAAACGGGT	1080
13	GCAAACACAGCAGCCTTACAG	TGTAGGGGGTTAAGCGGTG	1129
14	AACCGCCCTAGCAGTTACAATC	TTGATGCTCCGTTTGCGTGTA	1207
15	TTGGATCCCTCCTAGGAATTTGC	GGTGCTGATAGTGAGGCTATGG	1149
16	GGAGGACAGCCAGTCGAAC	TGTGTGCTTGATACCTGCTCC	1667

**Table 2:** Analysis of maternal genetic diversity in 11 goat populations using whole mitochondrial DNA sequences.

Population	Individuals	H	Variable Site	HD ± SD	π	k
Dangjin (KD)	37	13	22	0.806 ± 0.046	0.00025	4.111
Jangsu (KJ)	54	15	41	0.678 ± 0.068	0.00022	3.646
Tong-yeong (KT)	51	17	41	0.608 ± 0.082	0.00033	5.360
Gyeong-Sang Uni. (KG)	2	2	1	1.000 ± 0.500	0.00006	1.000
Crossbred (KC)	17	10	47	0.897 ± 0.056	0.02061	10.088
Korean Species	161	39	68	0.659 ± 0.044	0.00031	5.012
Nubian	2	2	22	1.000 ± 0.500	0.00132	22.000
Alpine	6	6	103	1.000 ± 0.096	0.00323	53.733
Toggenburg	28	23	238	0.981 ± 0.016	0.00331	54.103
Saanen	37	28	187	0.980 ± 0.012	0.00207	34.474
Boer	48	38	150	0.948 ± 0.010	0.00154	25.607
Ref (GenBank)	82	81	981	1.000 ± 0.000	0.00357	59.291
Overall	364	203	1016	0.925 ± 0.012	0.00202	31.687

Abbreviations: H, haplotype; HD, haplotype diversity; SD, standard deviation; π, nucleotide diversity; k, average number of nucleotide differences.

## Data Availability

The data presented in this study are available on request from the corresponding author as the research project is conducted by a national institution.
